# The Role of Autologous Dermal Micrografts in Regenerative Surgery: A Clinical Experimental Study

**DOI:** 10.1155/2019/9843407

**Published:** 2019-09-08

**Authors:** Marco Mario Tresoldi, Antonio Graziano, Alberto Malovini, Angela Faga, Giovanni Nicoletti

**Affiliations:** ^1^Plastic and Reconstructive Surgery, Department of Clinical Surgical, Diagnostic and Pediatric Sciences, University of Pavia, Viale Brambilla, 74 Pavia, Italy; ^2^Plastic and Reconstructive Surgery Unit, Department of Surgery, Istituti Clinici Scientifici Maugeri, Via Salvatore Maugeri, 10 Pavia, Italy; ^3^Department of Public Health, Experimental and Forensic Medicine, University of Pavia, Via Forlanini 6, Pavia, Italy; ^4^Sbarro Health Research Organization (SHRO), Temple University, 1900 N 12th St., Philadelphia, PA 19122, USA; ^5^Laboratory of Informatics and Systems Engineering for Clinical Research, Istituti Clinici Scientifici Maugeri, Via Salvatore Maugeri, 10 Pavia, Italy; ^6^Advanced Technologies for Regenerative Medicine and Inductive Surgery Research Center, University of Pavia, Viale Brambilla, 74 Pavia, Italy

## Abstract

The aim of the study was the objective assessment of the effectiveness of a microfragmented dermal extract obtained with Rigenera™ technology in promoting the wound healing process in an *in vivo* homogeneous experimental human acute surgical wound model. The study included 20 patients with 24 acute postsurgical soft tissue loss and a planned sequential two-stage repair with a dermal substitute and an autologous split-thickness skin graft. Each acute postsurgical soft tissue loss was randomized to be treated either with an Integra® dermal substitute enriched with the autologous dermal micrografts obtained with Rigenera™ technology (group A—Rigenera™ protocol) or with an Integra® dermal substitute only (group B—control). The reepithelialization rate in the wounds was assessed in both groups at 4 weeks through digital photography with the software “ImageJ.” The dermal cell suspension enrichment with the Rigenera™ technology was considered effective if the reepithelialized area was higher than 25% of the total wound surface as this threshold was considered far beyond the expected spontaneous reepithelialization rate. In the Rigenera™ protocol group, the statistical analysis failed to demonstrate any significant difference vs. the controls. The old age of the patients likely influenced the outcome as the stem cell regenerative potential is reduced in the elderly. A further explanation for the unsatisfying results of our trial might be the inadequate amount of dermal stem cells used to enrich the dermal substitutes. In our study, we used a 1 : 200 donor/recipient site ratio to minimize donor site morbidity. The gross dimensional disparity between the donor and recipient sites and the low concentration of dermal mesenchymal stromal stem cells might explain the poor epithelial proliferative boost observed in our study. A potential option in the future might be preconditioning of the dermal stem cell harvest with senolytic active principles that would fully enhance their regenerative potential. This trial is registered with trial protocol number NCT03912675.

## 1. Introduction

The dermal extracellular matrix plays a relevant both structural and functional role in signalling cell proliferation, development, shaping, function, and migration. The dermis is provided with a relevant both mesenchymal and adnexa-related stem cell pool. Such properties support the use of dermis-derived extracts to stimulate tissue regeneration [1–3]. Currently, many technologies are available to separate and expand dermis-derived cells to obtain injectable autologous cell suspensions for regenerative purposes [4, 5]. Recently, in the European Union area, cell manipulation underwent restricting regulations that significantly reduced the availability of cell expansion technology. According to current regulations, any cell manipulation involving enzymatic treatment and cell culture expansion is allowed in Cell Factories only, with a relevant increase of time and cost burden [6].

Recently, the development of an innovative technology for dermal mechanical microfragmentation named Rigenera™ allowed the harvest of a filtered available cell pool without any enzymatic manipulation. Such a cell fraction, rich in progenitor cells, was successfully used in the treatment of difficult-to-heal wound [3, 7–9]. The advantage of this innovative technology is its unrestricted use in any clinical context and setting.

Nevertheless, such an evidence was demonstrated within the frame of pathologies with heterogeneous aetiology. The aim of the study was the objective assessment of the effectiveness of such a microfragmented dermal extract in promoting the wound healing process in an *in vivo* homogeneous experimental human acute surgical wound model.

## 2. Materials and Methods

### 2.1. Study Design

A prospective randomized controlled open clinical trial was carried out at the Plastic and Reconstructive Surgery Unit of the Istituti Clinici Scientifici Maugeri. Twenty patients (4 females and 16 males), with an age range of 53-93 years (mean 77.80, median 79), were enrolled in the trial over a period of 15 months, from September 2017 to December 2018. The exclusion criteria were wound infection, chemotherapy in the last 6 months, use of corticosteroids or immunosuppressive treatment, and metabolic, endocrine, autoimmune, and collagen diseases. The study included patients with a postsurgical defect in any site of the body with a size range of 4-400 cm^2^. The surgical defect followed an immediate excision of 22 skin cancers, 1 ulcerated actinic keratosis, and 1 chronic difficult-to-heal wound (Table 1). A sequential two-stage repair with a dermal substitute and an autologous split-thickness skin graft was planned. The acute postsurgical soft tissue loss was considered the experimental unit of the study irrespective of the number of wounds per patient. Twenty-four experimental units were enrolled in the trial. Each unit, which fulfilled the entry criteria, was randomized to be treated either with an Integra® dermal substitute enriched with the autologous dermal micrografts obtained with Rigenera™ protocol (group A—Rigenera™ protocol) or with an Integra® dermal substitute only (group B—control). Each group included 12 experimental units. All of the wounds were planned for a sequential second-stage repair with a split-thickness skin graft at the time of complete engraftment of the Integra® dermal substitute. According to our clinical experience, the time lag between the first and the second surgical stages was around 4 weeks. The expected endpoint was a spontaneous reepithelialization higher than 25% of the total wound area in the group treated with Rigenera™ protocol at 4 weeks that would contraindicate the second staged cover with a split-thickness skin graft. The secondary endpoint was the comparison of the reepithelialization rate at 4 weeks after the first surgical stage between the group treated with Rigenera™ protocol and the controls.

**Figure 1:**
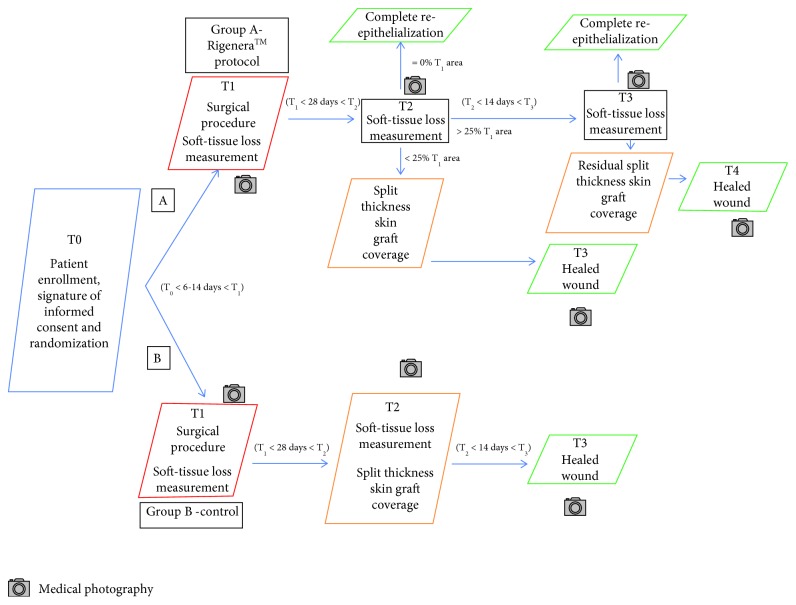
Scheme of the overall study structure.

The reepithelialization rate in the wounds was assessed at each time point of the study through digital photography with the software “ImageJ” (Figure 1). As a wound spontaneously reduces in size, due to a physiologic shrinkage process, the measurement of the reepithelialization rate was referred to the actual total wound size at each time point.

A formal informed written consent was obtained from all of the patients, and the study conformed to the Declaration of Helsinki. The trial was approved by the Ethical Committee (protocol number 2142) of the Istituti Clinici Scientifici Maugeri SB SpA IRCCS of Pavia.

### 2.2. Materials and Methods

The micrografts were obtained by Rigeneracons, a single-use sterile CE-certified Class I medical device able to mechanically disaggregate tissues into micrografts that are immediately available for transfer in the clinical practice [10]. It is made of a plastic container provided with an openable lid divided into two chambers by a stationary stainless steel grid with 100 hexagonal holes. Around each hole, 6 microblades are designed for efficient cutting of hard and soft tissues allowing a filtration cut-off of about 80 *μ*m. The upper chamber is provided with a rotating helix forcing the tissue fragments through the grid towards the bottom chamber. The rotation of Rigeneracons is activated by a Rigenera OR-PRO machine (Esacrom, Italy) using a connection adaptor (Adacons Max).

### 2.3. The Operative Protocol

**Figure 2:**
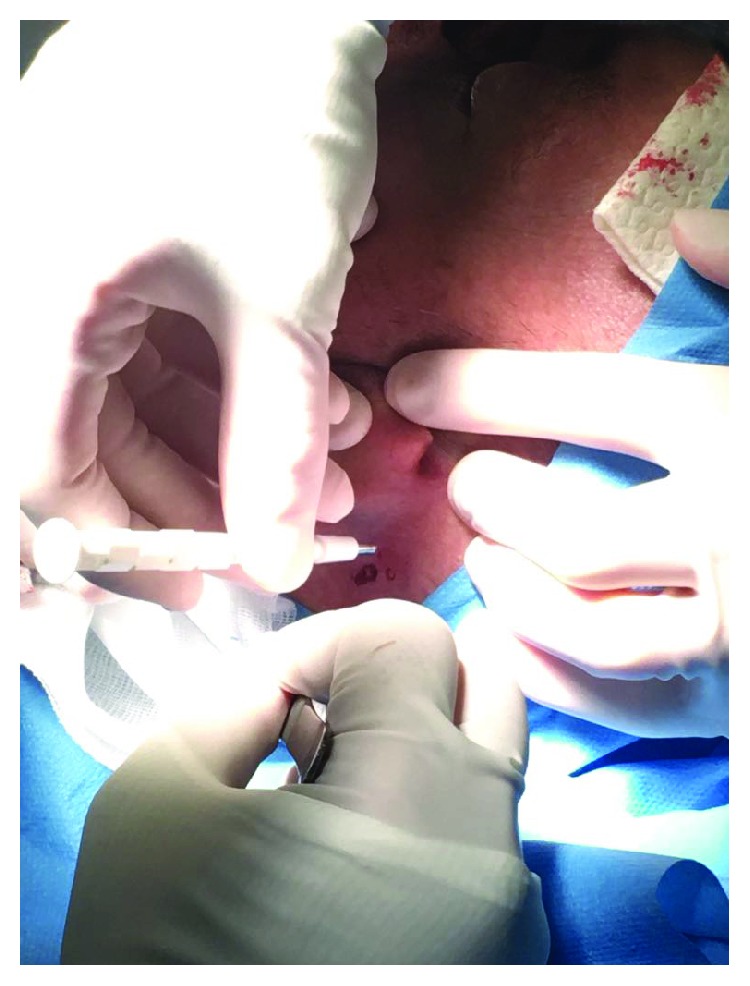
Harvest of the 3 mm punch biopsies from the previously deepithelialized skin.

**Figure 3:**
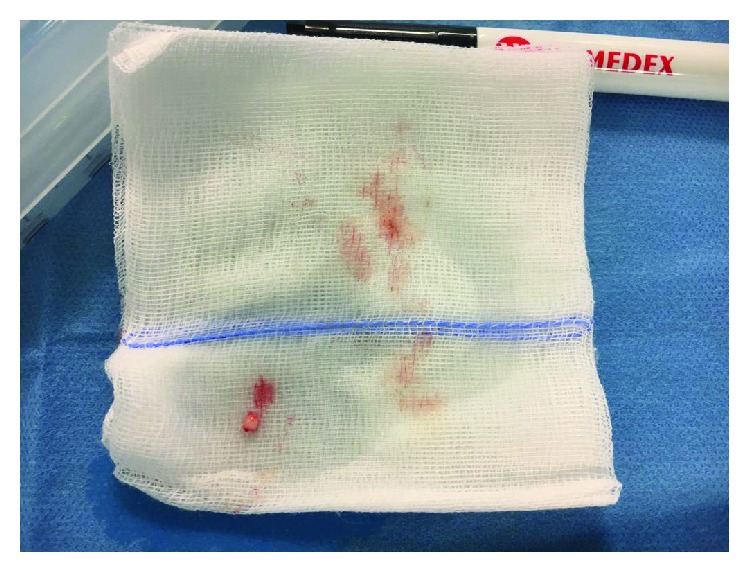
The harvested skin punch biopsy.

**Figure 4:**
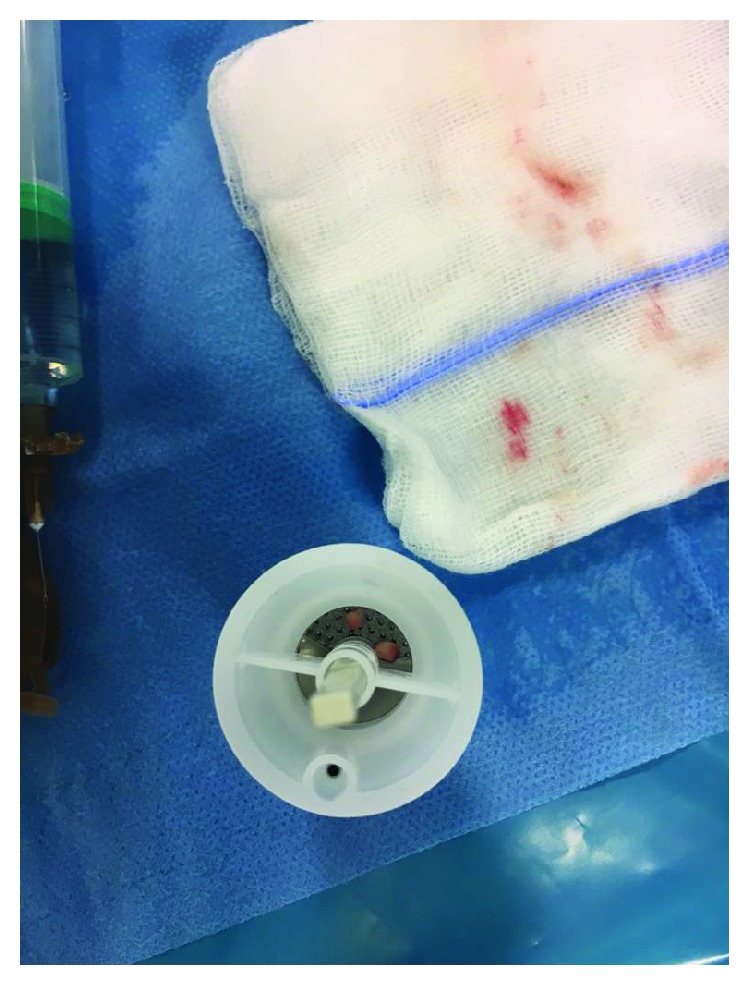
The disposable Rigeneracons loaded with a maximum of 3 dermal samples.

**Figure 5:**
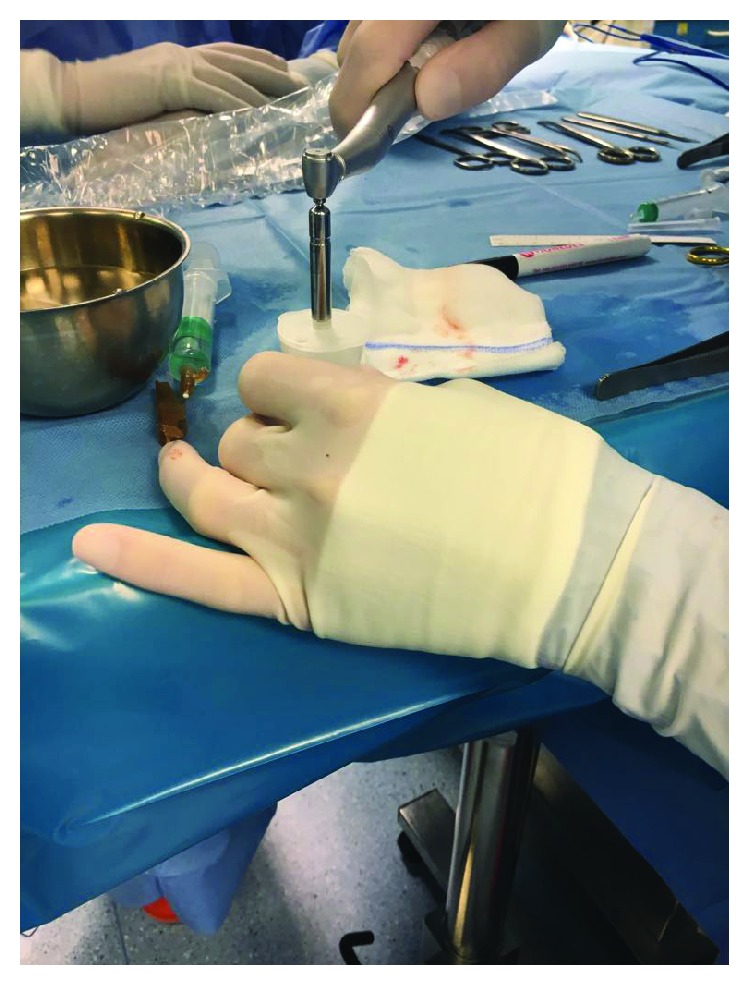
Connection of the disposable Rigeneracons to the Rigenera OR-PRO rotating machine.

**Figure 6:**
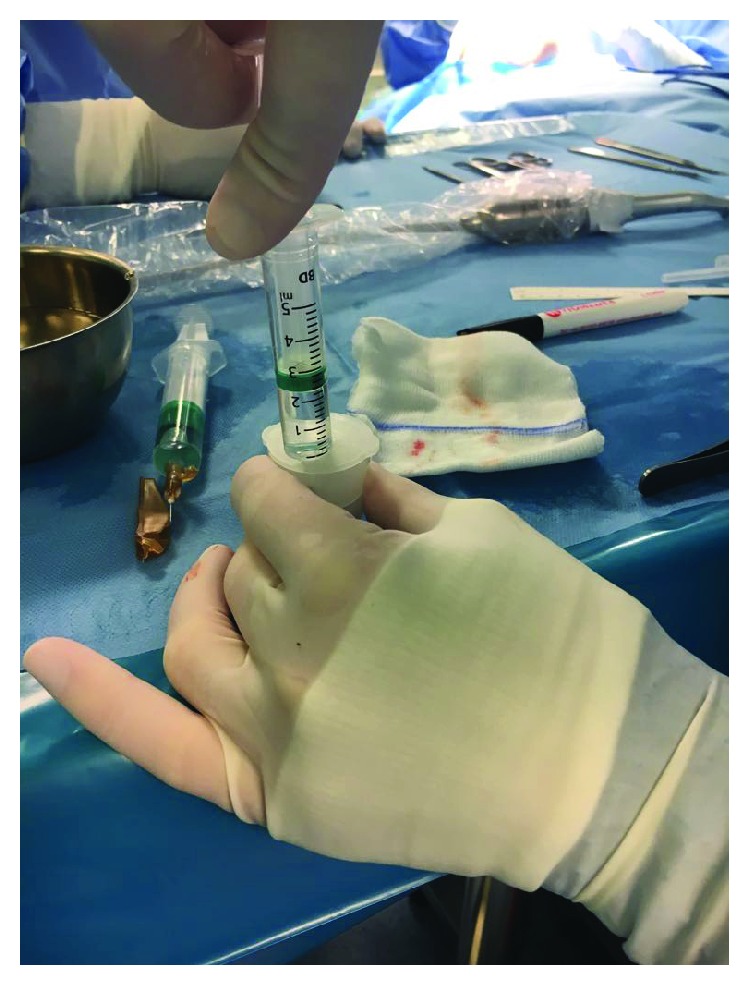
Aspiration of the micrografts containing saline solution with a sterile syringe.

**Figure 7:**
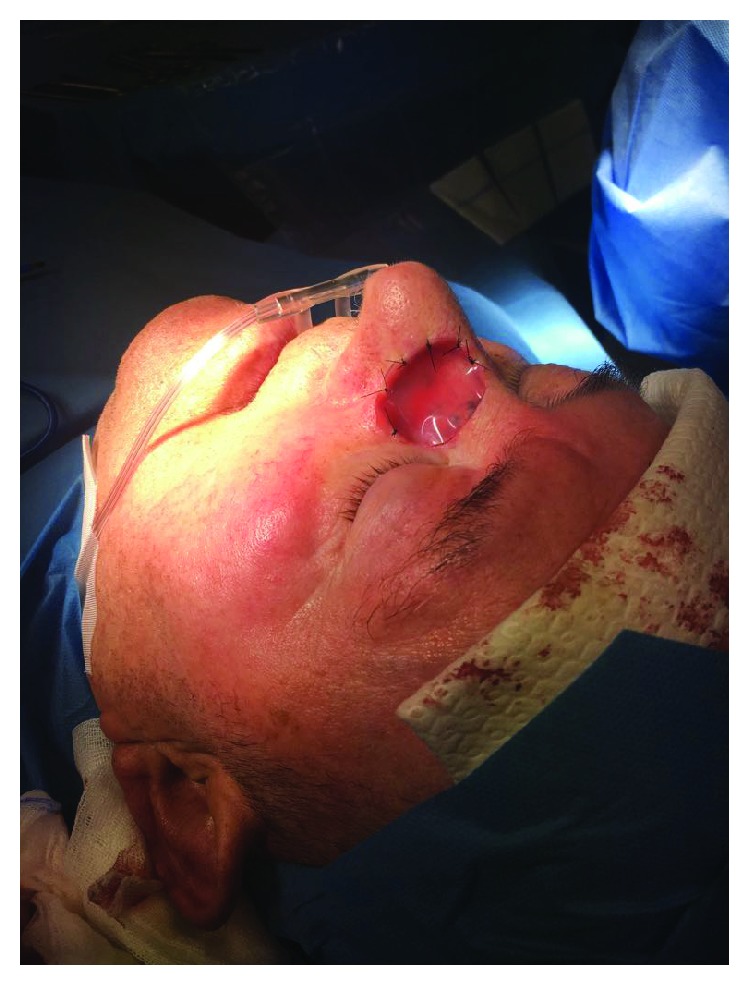
Cover of the postsurgical soft tissue loss with Integra® dermal substitute.

**Figure 8:**
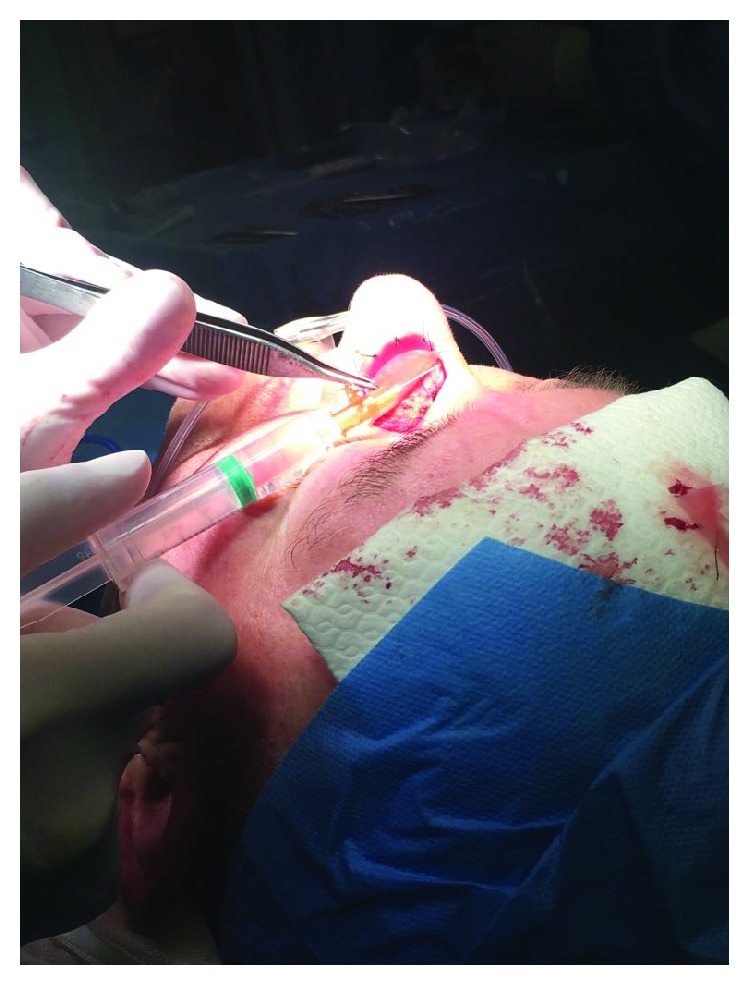
Imbibition of the dermal substitute with the saline micrograft suspension.

The operative protocol consists of different steps:

- Choice of the micrograft donor site with preference of the retroauricular region
- Gentle blade shaving of the donor site to remove the epidermis and obtain a bare papillary dermis
- Harvest of the adequate number of 3 mm punch biopsies from the previously deepithelialized skin; the number of dermal punch biopsies calculated according to the size of the wound, considering that 1 mm^2^ of the dermal graft was expected to regenerate an epithelial cover up to 2 cm^2^ (Figure 2)
- Loading the disposable Rigeneracons with a maximum of 3 dermal samples at a time and addition of 2.5 ml of saline solution (Figures 3 and 4)
- Device connection to the rotating machine, operating at 80 rpm for 90 seconds, that provides a mechanical disaggregation of the dermal sample into a suspension containing autologous dermal micrografts (Figure 5)
- Aspiration of the micrografts containing saline solution with a sterile syringe (Figure 6)
- Cover of the postsurgical soft tissue loss with Integra® dermal substitute (Figure 7)
- Fixation of the dermal substitute with stitches and gentle imbibition with the saline micrograft suspension (Figure 8)
- Infiltration of the micrograft suspension in the perilesional tissues

### 2.4. The Treatment

The time points of the study were designed as follows:

- T_0_: starting time includes patient enrollment, signature of informed consent, and randomization.
- T_1_: the first-stage was characterized by surgical procedure of skin lesion excision, digital medical photographs, and soft-tissue loss measurement were obtained with the use of the “ImageJ” program. Soft tissue loss was covered with an Integra® dermal substitute alone (group B—controls) or enriched with the autologous dermal micrografts (group A—Rigenera™ protocol).
- T_2_: 4 weeks after the first surgical stage, digital medical photographs and residual soft-tissue loss measurement were obtained with the use of the software “ImageJ.” In the Rigenera™ protocol group, if the deepithelialized area in the wound was the same as at T_1_, a split-thickness skin graft was planned; if the reepithelialized area was >25% than the one at T_1_, a follow-up was planned in 2 weeks' time (T_3_); if reepithelialization was complete, the wound was considered healed and the patient was discharged from the study. In the control group, a split-thickness skin graft cover was carried out.
- T_3_: digital photographs and residual soft-tissue loss measurement were obtained with the use of the software “ImageJ” in the Rigenera™ protocol group; in the latter group, whatever the extension of the residual deepithelialized area, a split-thickness skin graft was planned at this time; if reepithelialization was complete, the wound was considered healed and the patient was discharged from the study.
- T_4_: there was complete reepithelialization 1 week after the split-thickness skin graft cover at T_3_ in the Rigenera™ protocol group.

### 2.5. Statistical Methods

The deviation of continuous variable distribution from the normal distribution was assessed by visual inspection of quantile-quantile plots and by the Shapiro test. T1 and final area distribution was normalized by natural logarithm transformation. Continuous variable distribution is described by median and 25^th^–75^th^ percentiles; categorical variable distribution is described by counts and frequencies (%). The Fisher exact test and the Wilcoxon rank-sum test were applied to compare the categorical and quantitative variables' distribution between protocols. The Spearman test allowed quantifying the strength of the correlation between continuous variables (rho). Statistical procedures were performed by the R statistical software (http://www.r-project.org.)

## 3. Results

One male patient out of 20 with surgical excision of a squamous cell carcinoma of the scalp dropped out of the study due to postoperative wound infection. An overall of 23 experimental units (12 in the control group and 11 in the Rigenera™ protocol group) completed the trial.

At T_2_, 4 weeks after the first surgical step, the reepithelialization rate was 12.98% (10.40-17.61) in the control group and 15.14% (12.42-22.03) in the Rigenera™ protocol group (*p* = 0.607). In the latter group, only one wound out of 11 (9.09%) demonstrated a reepithelialization > 25% of the total wound area, while in the control group, such an outcome was observed in 2 wounds out of 12 (16.67%) (*p* = 1).

## 4. Discussion

It is a common knowledge that the human dermis is a source of stem cells with demonstrated regenerative properties [11–16]. The dermal mesenchymal stromal stem cells display adhesion properties, fibroblast morphology, and osteogenic and adipocyte differentiation. Typically, they express both mesenchymal (*α*-SMA) and neural (Nestin and *β*III-tubulin) cell membrane markers and lack the haematopoietic and endothelial ones (CD31) [13]. Recently, the dermal mesenchymal stromal stem cells were demonstrated to express also the CD105, CD73, and CD90 markers that specifically regulate regeneration in the wound healing process. These cells enhance cell survival and proliferation in the wound site through a fine modulation of the immune and inflammatory response, operated by a finely tuned cascade of local mediators. They definitely play a relevant active role along the inflammatory, proliferative, and remodeling phases allowing an eventual favorable outcome in the wound healing process.

The hair follicle matrix has been demonstrated to host cells that are capable of self-renewal and produce epithelial transient progenitors, thus having attributes of stem cells, too. Stem cells are multipotent, capable of giving rise not only to all the cell types of the hair but also to the epidermis and the sebaceous gland. These cells display a highly sophisticated organization and carry out several functions controlling the shape of the hair follicle. The inner structures are each produced by a distinct, restricted set of precursors occupying a specific position along the proximodistal axis of the matrix. The matrix seems to be organized by two systems working in orthogonal dimensions and controlling two key operations of hair follicle morphogenesis, notably cell diversification and cell behavior [17]. The bulge cell progeny located in the upper follicle has been demonstrated to emigrate into the epidermis and to proliferate, thus contributing to the long-term maintenance of the epidermis [18, 19]. Based on these observations, it has been proposed that the bulge is a major repository of skin keratinocyte stem cells, which may thus be regarded as the ultimate epidermal stem cell [20, 21]. Since stem cells are known to be involved in skin tumor formation [22–27], the coincidence of greater tumor susceptibility with the transient proliferation of the bulge cells is consistent with the hypothesis that the bulge cells are stem cells and indicates that follicular stem cells can give rise to experimentally induced skin cancers [22–25]. Taken together, these data suggest that the bulge is the site of follicular stem cells.

The Rigenera™ method allows the harvesting of a dermis-derived autologous cell suspension including a stem cell fraction, ready for use without any cell manipulation process [28]. Several clinical studies demonstrated the effectiveness of the Rigenera™ cell harvesting method in the management of complex wounds with complete obliteration and reepithelialization of deep soft tissue loss [1–3, 7–9].

In order to objectively assess the effectiveness of the Rigenera™ method, we established a homogeneous experimental fresh surgical wound model providing measurable data that excluded gross experimental bias and fit a rigorous statistical analysis.

Rigenera™ provides a fluid cell suspension that may be both injected in deep spaces and applied on superficial soft tissue loss in combination with a dermal substitute [29].

According to our current clinical practice, we deliberately enrolled patients with a planned two-stage soft tissue loss repair using a dermal substitute followed 4 weeks later by a split-thickness skin graft. The enrichment of a dermal substitute with an autologous cell suspension graft was considered a minimal modification of a current and well-established clinical protocol involving a negligible donor site morbidity and, therefore, allowed approval of the trial by the Ethical Committee.

Considering our long-term clinical experience in the field [30], the dermal substitute of choice for the study was Integra® as it was demonstrated to provide an in vitro favorable environment for dermal stromal mesenchymal stem cell engraftment and replication as early as 7 days [29]. The peculiar three-dimensional structure of Integra®, with a controlled 80 *μ*m porous structure, allows a physiological cell adhesion, infiltration, distribution, and proliferation with the preservation of the typical mesenchymal fibroblast morphology.

In our study, the treatment with the dermal cell suspension prepared with the Rigenera™ technology was considered effective if the reepithelialized area was higher than the 25% of the total wound surface as the latter threshold was considered far beyond the expected spontaneous reepithelialization rate.

Nevertheless, in the Rigenera™ protocol group, the statistical analysis did not demonstrate any significant difference vs. the controls.

The old age of the patients likely influenced the outcome.

**Figure 9:**
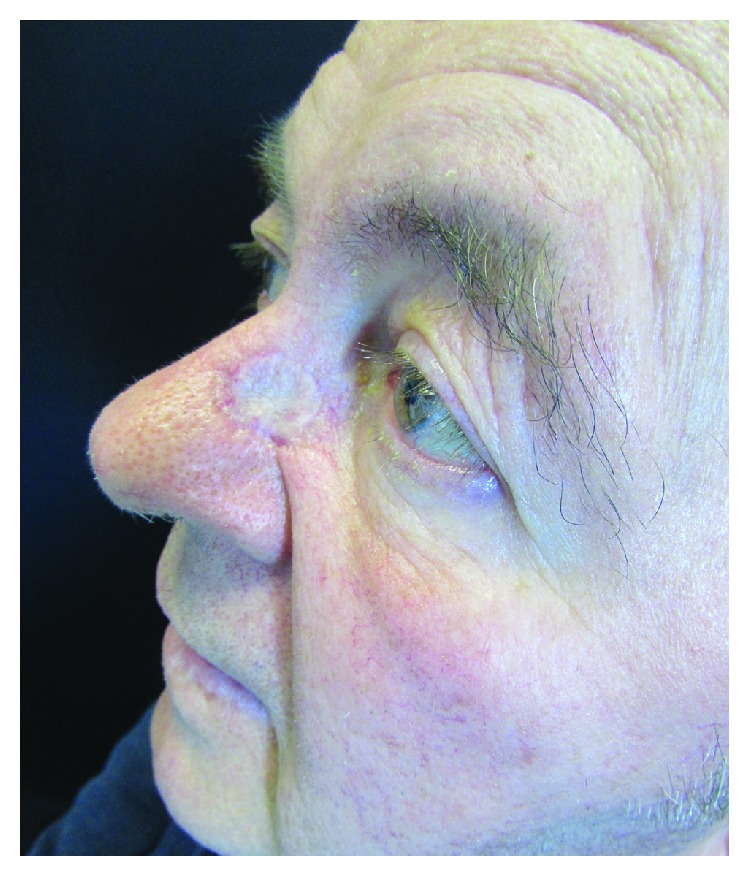
Complete healing of the defect after STSG in group A.

Indeed, our experimental plan, designed as a two-stage procedure, had to meet ethical requirements, too. Currently, such a procedure is the gold standard in frail elderly patients that often come to observation for advanced skin cancers, requiring extensive demolitions but that are unfit for complex reconstructive procedures [31]. The use of a sequential two-stage reconstructive procedure with a dermal substitute and a split-thickness skin graft in these cases allows for a better functional and cosmetic outcome than a simple one-staged skin graft [32–34] (Figure 9).

Undoubtedly, the stem cell regenerative potential is reduced in the elderly. Recent literature reports demonstrate an antiapoptotic action of the senescent cells that prevents the full expression of the regenerative potential in the stem cell pool [35]. In our opinion, a sample pretreatment with specific active principles targeting the senescent cells might be suggested to increase the regenerative potential in the dermal stromal mesenchymal stem cell transfer [36]. The latter specific pretreatment might enhance the full regenerative potential of a minimally invasive cell transfer, making it a specifically convenient procedure for the frail critical patient. Therefore, reepithelialization of large skin loss in the elderly patient drawing on a minimal dermal fragment might be a realistic option.

A further explanation for the unsatisfying results of our experimental trial might be the inadequate amount of dermal stem cells used to enrich the dermal substitutes. Actually, in our study, we used a 1 : 200 donor/recipient site ratio in order to minimize donor site morbidity. The gross dimensional disparity between the donor and recipient sites and the low concentration of dermal mesenchymal stromal stem cells might explain the poor epithelial proliferative boost observed in our study. Unpublished data from animal experimental trials by our research partner staff would suggest that the optimal donor/recipient site ratio is 1 : 20. Nevertheless, such a ratio would not make the dermal cell suspension transfer a convenient procedure in terms of donor site morbidity vs. a traditional large meshed split-thickness skin graft [37].

Actually, in previous literature reports, the Rigenera™ dermal cell transfer proved to be effective in the difficult-to-heal wound where a split-thickness skin graft was not indicated. Therefore, we suppose that the reported favorable outcome might have been related to an overall change of the wound environment, where a spontaneous reepithelialization might have been related to a nonspecific boost of a torpid wound bed from mesenchymal dermal and epithelial stem cells and matrix-derived factors. Instead, in our opinion, in an acute fresh wound, all of the factors involved in the wound healing process display a maximal expression, thus shading the supposed contribution of the dermal extract as a whole. Indeed, a preconditioning of the dermal cells with a treatment enhancing their regenerative potential might yield a better outcome.

## 5. Conclusions

The role of the human dermal stem cell regenerative pool in enhancing the wound healing process is a well-established knowledge and is leading to an increasing number of promising clinical applications. The Rigenera™ technology might promote a spontaneous reepithelialization; nevertheless, even if proved to be effective in stimulating a difficult-to-heal wound by turning a torpid chronic process into an active one, in our experience, it could not demonstrate any improvement in the reepithelialization process of a fresh surgical wound. A potential option in the future might be a preconditioning of the dermal stem cell harvest with senolytic active principles that would fully enhance their regenerative potential. Such a treatment might extend the clinical indications of this minimally invasive, standardized, operator-independent, and easy procedure, specifically suitable for the management of complex and critical cases.

## Figures and Tables

**Table 1:** Cohort's characteristics. Categorical variables' distribution is described by counts and relative frequencies (%); continuous variables' distribution by median (25^th^–75^th^ percentiles).

Variable	Distribution
Age (years)	78.0 (74.5-84.0)
Gender	
Females	4 (17.39%)
Males	16 (82.61%)
Localization	
Limbs	7 (30.43%)
Scalp	1 (4.35%)
Face	15 (65.22%)
Protocol	
A	11 (47.83%)
B	12 (52.17%)
Cause	
BCC	16 (69.57%)
SCC	4 (17.39%)
Other	3 (13.09%)
T1 area (cm^2^)	9.26 (7.06-12.54)
Reepithelialization (%)	13.94 (11.96-20.85)
≥25%	3 (13.04%)
<25%	20 (86.96%)

## Data Availability

The data used to support the findings of this study are restricted by the Ethical Committee (protocol number 2142) of the Istituti Clinici Scientifici Maugeri SB SpA IRCCS of Pavia in order to protect patient privacy. Data are available for researchers who meet the criteria for access to confidential data from the corresponding author upon request.
